# Multilocus Sequence Analysis for *Leishmania braziliensis* Outbreak Investigation

**DOI:** 10.1371/journal.pntd.0002695

**Published:** 2014-02-13

**Authors:** Mariel A. Marlow, Mariana C. Boité, Gabriel Eduardo M. Ferreira, Mario Steindel, Elisa Cupolillo

**Affiliations:** 1 Laboratório de Protozoologia, Departamento de Microbiologia, Imunologia e Parasitologia, Universidade Federal de Santa Catarina, Florianópolis, Santa Catarina, Brazil; 2 Laboratório de Pesquisa em Leishmaniose, Instituto Oswaldo Cruz, Fundação Oswaldo Cruz, Rio de Janeiro, Brazil; Charité University Medicine Berlin, Germany

## Abstract

With the emergence of leishmaniasis in new regions around the world, molecular epidemiological methods with adequate discriminatory power, reproducibility, high throughput and inter-laboratory comparability are needed for outbreak investigation of this complex parasitic disease. As multilocus sequence analysis (MLSA) has been projected as the future gold standard technique for *Leishmania* species characterization, we propose a MLSA panel of six housekeeping gene loci (6*pgd*, *mpi*, *icd*, *hsp*70, *mdh*mt, *mdh*nc) for investigating intraspecific genetic variation of *L.* (*Viannia*) *braziliensis* strains and compare the resulting genetic clusters with several epidemiological factors relevant to outbreak investigation. The recent outbreak of cutaneous leishmaniasis caused by *L.* (*V.*) *braziliensis* in the southern Brazilian state of Santa Catarina is used to demonstrate the applicability of this technique. Sequenced fragments from six genetic markers from 86 *L.* (*V.*) *braziliensis* strains from twelve Brazilian states, including 33 strains from Santa Catarina, were used to determine clonal complexes, genetic structure, and phylogenic networks. Associations between genetic clusters and networks with epidemiological characteristics of patients were investigated. MLSA revealed epidemiological patterns among *L.* (*V.*) *braziliensis* strains, even identifying strains from imported cases among the Santa Catarina strains that presented extensive homogeneity. Evidence presented here has demonstrated MLSA possesses adequate discriminatory power for outbreak investigation, as well as other potential uses in the molecular epidemiology of leishmaniasis.

## Author Summary

Molecular epidemiology of infectious diseases, which uses pathogen genetics to determine risk factors in the human population, is commonly employed to assist in outbreak investigation. While definitive genetic markers and techniques have been developed for several other bacterial, viral, and parasitic pathogens, the scientific community has yet to agree on an international standard for inter- and intra-species differentiation of *Leishmania*, the parasite that causes the disease leishmaniasis. As leishmaniasis represents one of the highest disease burdens among the neglected tropical diseases, development of molecular techniques, which allow for inter-laboratory comparability through international sequence databases, is imperative for moving forward with disease control. Based on the current standard technique employed for bacteria, the authors propose a panel of six genetic markers for multilocus sequence analysis (MLSA) for intraspecific differentiation of *Leishmania braziliensis*, the most widely distributed of the *Leishmania* species in Brazil. Using strains from a recent outbreak in the sub-tropical non-endemic southern Brazil in comparison with strains from eleven other Brazilian states, the authors provide a practical example of how this technique can be applied in a real world outbreak situation.

## Introduction

Leishmaniasis, a vector-borne disease caused by protozoan parasites of genus *Leishmania* [1], represents one of the highest disease burdens among the neglected tropical diseases in developing nations [2]. While not often fatal like the visceral form, the cutaneous form of the disease contributes substantially to leishmaniasis disease burden as it requires a lengthy and costly treatment regimen, results in apparent scarring, and can progress to a severely disfiguring mucosal form [1]. In recent years, leishmaniasis outbreaks have been described with increasing frequency [3]–[5], including those in sub-tropical regions or regions not previously endemic across the global [6]–[8]. In Brazil, beginning in 2005, an outbreak of human cutaneous leishmaniasis occurred in the southern Brazilian state of Santa Catarina, where the disease had not been observed previously as endemic. Overtime, cutaneous leishmaniasis has emerged in the region with evidence of a continued transmission cycle [9]. The species responsible for this outbreak has been incriminated as *Leishmania* (*Viannia*) *braziliensis* [9], the most widely distributed *Leishmania* species in Brazil to date [10], [11]. However, many questions still remain regarding the outbreak, such as: is one main strain or various strains responsible for the outbreak; is the emergence of *L.* (*V.*) *braziliensis* in the region a recent event; and how are Santa Catarina strains related to other strains in Brazil? A wide range of molecular tools are available for the investigation of molecular epidemiology of leishmaniasis, but choosing which method and/or markers to use continues to be a challenge [12]. Particularly for New World species, open access databases based on gold-standard genetic markers have not been developed. Currently, outbreak investigation of leishmaniasis, mainly conducted for visceral leishmaniasis outbreaks caused by *L.* (*Leishmania*) *donovani* species complex [13], [14], commonly employs multilocus microsatellite typing (MLMT). This technique has been proven to discriminate at the intra-species level [15] with high discriminatory power and is useful for determining outbreak strain origin when a database of MLMT strains is available for the *Leishmania* species of interest [13]. At the present moment, an open access MLMT database for *L.* (*V.*) *braziliensis*, has not been developed. The high discriminatory power of this technique has its drawbacks depending on the type of epidemiological question or analysis. In some cases, almost 20 “different” genotypes can be identified in one focus [13], [16], [17]. Dividing the isolates into many different genotypes reduces the statistical power of analyses involving epidemiological variables, such as clinical and demographic characteristics of the patient. Such reductions in statistical power greatly reduce the ability of researcher to conclude the relationship of factors like clinical form and disease virulence with a particular genotype.

Thus, epidemiological tools with appropriate discriminatory power, increased reliability and inter-laboratory reproducibility and comparability urgently are required. With these characteristics in mind, the method of multilocus sequence analysis (MLSA) provides a promising alternative. Projected as the future gold standard species typing method [12], MLSA involves sequencing a panel of house-keeping gene loci based on the panel of enzymes used in MLEE [18]. Several markers of these conserved regions have already been described, including ten markers for *L.* (*L.*) *donovani* [19], [20], and six markers for New World species [18], [21]. However, for *L.* (*Viannia*) species, these studies have mainly focused on interspecies discrimination and phylogenetic/taxonomic analysis and have employed only up to four markers. Given the challenges described above, we propose a panel of six gene loci, including three new markers described here for the first time, as an epidemiological tool for investigation of *L.* (*V.*) *braziliensis* outbreaks. In the present study, the recent outbreak in Santa Catarina is used to demonstrate the applicability of this technique in outbreak settings. The overarching objective of this work will be to generate interest in the community of leishmaniasis investigators to create an international sequence database based on these gene markers, as well as other markers from the original MLEE panel, for a more comprehensive and unified investigation into the distribution and epidemiological characteristics of *Leishmania* species.

## Methods

### Ethics statement

Ethical approval for the use of patient data and their respective sample was received from the UFSC Ethics Committee. CLIOC is a Depository Authority of the Ministry of the Environment [Fiel Depositária pelo Ministério do Meio Ambiente, MMA] (D.O.U. 05.04.2005). Following Resolution 21 (August 31, 2006 – CGEN/MMA), authorization was not required for usage of samples previously deposited in CLIOC since the samples were used for research purposes only and data were analyzed anonymously.

### Data and sample collection

*Leishmania* (*Viannia*) *braziliensis* strains from eleven Brazilian states (n = 53) were obtained from the *Leishmania* Collection of the Oswaldo Cruz Institute (Coleção de *Leishmania* do Instituto Oswaldo Cruz- CLIOC) in Rio de Janeiro, Brazil, and strains from Santa Catarina (n = 33) were obtained from the cryobank of the Laboratório de Protozoologia of the Universidade Federal de Santa Catarina (UFSC), Florianópolis, Santa Catarina, Brazil. Patient data from Santa Catarina used in this study were investigated as part of routine reportable disease surveillance and collection procedures have been previously described in [9]. Santa Catarina isolates were deposited in CLIOC and subjected to MLEE characterization, according to routine procedures employed by CLIOC.

### PCR amplification and sequencing

*Leishmania* promastigotes were cultured at 25°C in Schneider's medium supplemented with 20% heat-inactivated fetal bovine serum. DNA extraction was conducted using the Wizard DNA purification Kit (Promega, Madison, USA), according to manufacturer's instructions.

Amplification was performed for a panel of six housekeeping gene loci listed in Table 1. Primers and PCR conditions have been previously described for 6-*phosphogluconate dehydrogenase* (6*pgd*), *manose*-6-*phosphate isomerase* (*mpi*), *isocitrate dehydrogenase* (*icd*) [18] and for the *heat shock protein* 70 (*hsp*70) [22], [23]. Primers for mitochondrial *malate dehydrogenase* (*mdh*mt) and nuclear *malate dehydrogenase* (*mdh*nc) are described here for the first time. Both follow the reaction condition: for 50 µl, 0,2 mM of each primer, 100 mM Tris–HCl, pH 8.8; 500 mM KCl, 1% Triton X-100; 15 mM MgCl_2_, 0.25 mM deoxyribonucleotide triphosphate (dNTPs), 0.025 U FideliTaq/GoTaq polymerase and 50 ng DNA. Amplification conditions were 94°C for 2 min, followed by 34 cycles at 94°C for 30 s, 52°C for 30 s and 72°C for 1 min, with a final extension at 72°C for 5 min. PCR products were purified and subsequently sequenced with the same primers used in the PCR.

**Table 1:** Amplicon size, analyzed sequence fragment length and primer sequence of the target regions for the six loci studied.

Locus	Amplicon size (bp)	Analyzed sequence length (bp)	Primer sequence (5′-3′)
6-*phosphogluconate dehydrogenase* (6*pgd*)	836	666	CTCAAGGAACATGAGCACGA TTGTCCTTGACTTGCTCACG
*Mannose*-6-*phosphate isomerase* (*mpi*)	681	569	GGCAAGATGTATGCGGAGTT CTCCCCAGGAACCATCTGTA
*Isocitrate dehydrogenase* (*icd*)	1022	755	GAATCGGGAAGGAGATCACA CATCATAGCCCCAGAGAGGA
*Heat-shock protein* 70 (*hsp*70)	1022	896	GGACGAGATCGAGCGCATGGT TCCTTCGACGCCTCCTGGTTG
*Malate dehydrogenase* mitochondrial (*mdh*mt)	821	666	TGCCGACCTCTTCCATATTC GAGTGAGGTGCGTCTTCACA
*Malate dehydrogenase* nuclear (*mdh*nc)	1010	803	TCACAACCGCAACTACGA CTACTCACGATAACGGCAGA

Consensus sequences were obtained and edited in the software package Phred/Phrap/Consed Version: 0.020425.c (University of Washington, Seattle, WA, USA) and only those with Phred values above 20 were used as contigs. Analyzed sequence fragment lengths for each marker are provided in Table 1. Contigs of all strains were mounted and aligned in MEGA4 (Molecular Evolutionary Genetics Analysis version 4) [24]. Ambiguous sites were divided into two of the possible alleles for all markers using the PHASE algorithm in DnaSP5 [25].

### Determination of clonal complexes

Clonal complexes (CC) were defined through BURST analysis in the software eBURSTv3 [26]. The BURST algorithm identified groups of mutually exclusive genotypes associated with a MLSA population and the founding genotype sequence within each group. Then, the algorithm provided the predicted descent from the founding genotype for all other genotypes [26], [27]. For this analysis, criterion for CC formation was fixed at the most stringent level with at least five identical alleles for the six loci defining a CC. Sequences which were not able to be grouped into a clonal complex remained in the analysis as unique sequences.

### Analysis of population structure

Haploid sequences rebuilt from the PHASE algorithm in DNAsp containing homozygous and heterozygous alleles were imported into STRUCTURE 2.3.4 (University of Chicago, Chicago, IL, USA) to investigate the population structure of the 86 samples of *L.* (*V.*) *braziliensis* based on the six MLSA loci. Using a Bayesian statistical approach, STRUCTURE applies a model-based clustering method to infer population structure and assign individuals to clusters based on multilocus genotype data [28]. Genetically distinct clusters (*K*) are identified based on the frequency of alleles, attributing the fraction of each genotype for each sample. In STRUCTURE, runs were performed using a burn-in period of 200,000 iterations followed by 600,000 running iterations. Runs were repeated three times to obtain data suitable for estimating the value of Δ*K* (defined as the rate of variation of the log likelihood of data between successive values of *K*), which provides the most likely *K* value for the data to be used in STRUCTURE HARVESTER [29]. STRUCTURE HARVESTER generates graphs for the change in the log of k and calculation of Δ*K* of STRUCTURE results, which were compared for choosing the *K* that best fit the data. Next, CLUMPP version 1.1.2 [30] was employed to align the multiple replicate analyzes of the same data set. Hierarchical analysis of two to seven *K* clusters was performed to define the assignment of borderline strains.

Based on clusters found in STRUCTURE, we used Microsatellite Analyser (MSA) [31] to estimate F*_ST_* values and Genetic Data Analysis (GDA) version 1.1 [32] to calculate expected heterozygosity (H_e_), observed heterozygosity (H_o_), and inbreeding coefficient (F*_IS_*). Recombination analysis was performed in Recombination Detection Program (RDP) [33].

### Development of median-joining network

To view genetic relationships (phylogenetic network) among strains and differentiation provided by the six markers, the median-joining network was mounted in the program SplitsTree 4.0 [34]. The median-joining network was constructed using concatenated character nucleotide sequences with ambiguous sites for all loci and strains. Nodes of the network, representing individual or groups of strains, were labeled by size, color and/or year/location to reflect epidemiological variables associated with the patient from whom the strain was isolated.

### Statistical analysis and mapping

Associations between genetic and epidemiological variables were analyzed in Stata SE 13 (StataCorp LP, College Station, TX, USA). Chi-squared test, or Fisher's exact test when appropriate, was used to assess the relationships between categorical variables. Maps were created in ArcGIS 10 (ESRI, Redlands, CA, USA).

## Results

**Figure 1:**
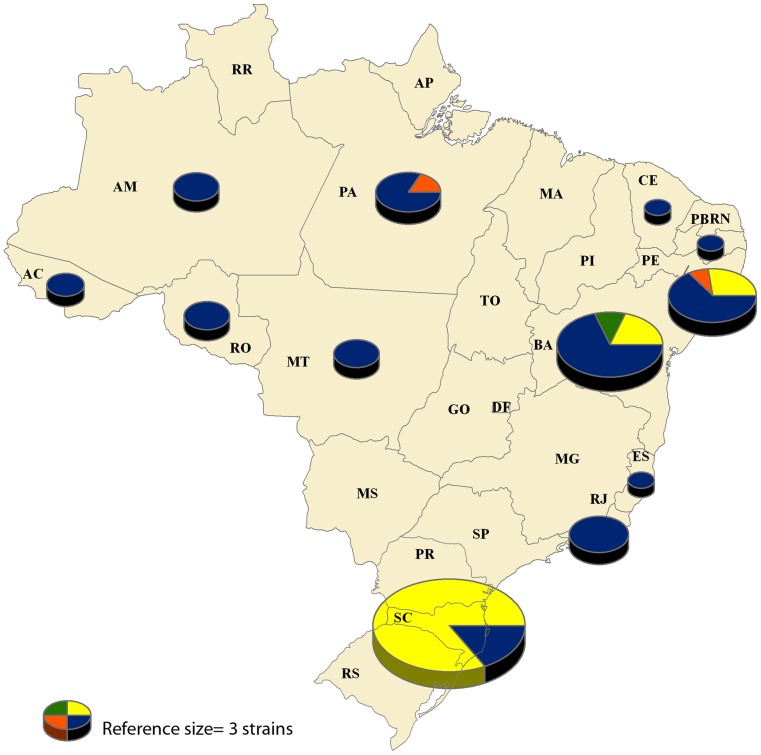
Geographical distribution of clonal complexes by state among Brazilian strains of *L.* (*V.*) *braziliensis* (n = 86). Colors represent clonal complex (CC) as follows: yellow, CC1; green, CC2; orange, CC3; blue, unique sequence types. The size of the individual pie charts was weighted according to number of strains from the state included in the analysis. A chart representing three samples is provided as a reference. State names are the same as described in Supporting Information S1.

BURST analysis identified three clonal complexes (CC) among the 86 strains of *L.* (*V.*) *braziliensis*, with over half (54.7%, 47/86) of the strains not belonging to any of the three CCs and remaining separate as unique sequence types (Supporting Information S1). A total of 76 distinct sequence types were observed among strains. The analysis was heavily weighted by the homogeneity and large number of strains from Santa Catarina included in the analysis, with the large majority (84.8%, 28/33) of Santa Catarina strains being grouped into one nearly exclusive clonal complex (CC1). Five out of six strains from Santa Catarina that did not group with CC1 were registered as imported cases in the epidemiological investigation. No association was found between CC and clinical form (p = 0.660). Figure 1 shows the geographical distribution of the CCs by state, revealing proportionally higher genetic variation in states from the Amazon biome (94.1% (n = 16/17) unique sequence types) (Supporting Information S1).

**Figure 2:**
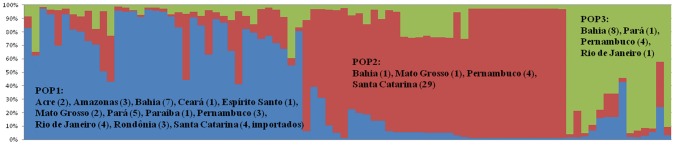
STRUCTURE output estimating genetic structure of the *L.* (*V.*) *braziliensis* strains from twelve Brazilian states (n = 86, *K* = 3). POP1 – Cluster 1; POP2 – Cluster 2; POP3 – Cluster 3; State (number of strains).

Through calculation of Δ*K* in the STRUCTURE analysis, the *L.* (*V.*) *braziliensis* strains included in the present study from 12 Brazilian states were found to best fit into three clusters (POP) (Supporting Information S1). Overall, 41.9% (36/86) of strains belonged to POP1, 40.7% (35/86) to POP2, and 16.3% (14/86) to POP3. As in the BURST analysis, the large majority (87.9%, 29/33) of Santa Catarina strains formed their own cluster (POP2), which also included four strains from Pernambuco, one from Mato Grosso and one from Bahia (Figure 2). The four Santa Catarina strains that did not cluster with POP2 were registered as imported cases in the epidemiological analysis. These four strains were the same strains from imported cases that did not cluster in the BURST analysis. Complete strain information can be found in Supporting Information S2.

**Figure 3:**
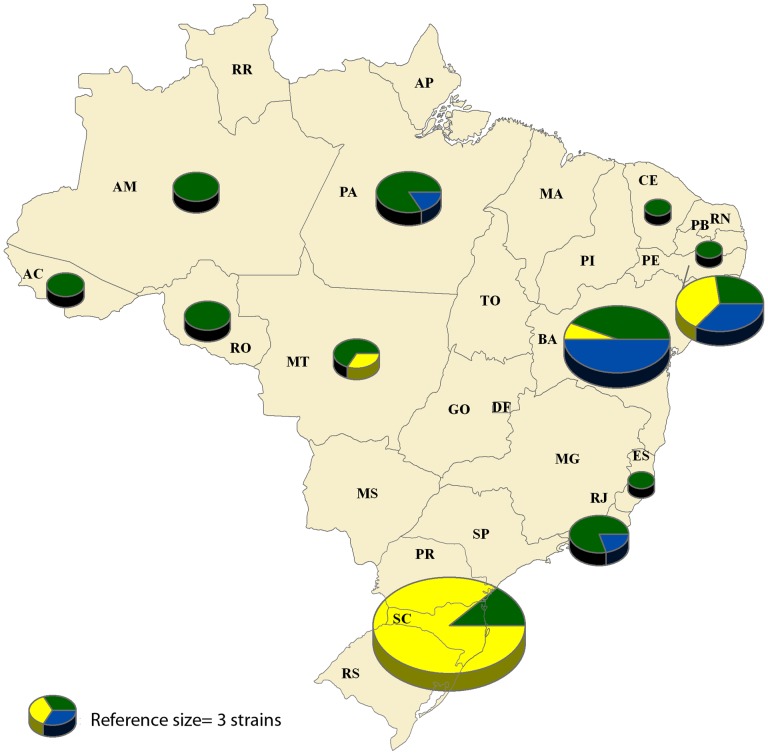
Geographical distribution of genetic clusters of Brazilian *L.* (*V.*) *braziliensis* strains as designated by STRUCTURE by state (*K* = 3, n = 86). Colors represent genetic clusters (POP) as follows: green, POP1; yellow, POP2; blue, POP3. The size of the individual pie charts was weighted according to number of strains from the state included in the analysis. A chart representing three samples is provided as a reference. State names are the same as described in Supporting Information S1.

As shown in Figure 3, POP1 demonstrated the most extensive geographical distribution, including strains from all states analyzed in this study. A distinction can be made between the genetic variation and genetic structure of coastal states, which contain Atlantic forest, and northern states, which are located in the Amazon basin. States of the Amazon region were predominately comprised of POP1 strains, while strains of POP2 and POP3 were mainly found in coastal states.

A significant association between the genetic cluster designated by STRUCTURE and leishmaniasis clinical form of the patient from which the strain was isolated was observed (p = 0.030) (Table 2). Most strains from cases presenting the mucocutaneous clinical form (4/7) belonged to POP3, including one case from Rio de Janeiro State, one from Pernambuco and two from Bahia.

**Table 2:** Leishmaniasis clinical form of patient by genetic cluster as attributed by STRUCTURE analysis of 86 *L.* (*V.*) *braziliensis* strains from Brazil.

	Cluster			
Clinical form	POP1	POP2	POP3	Total
Cutaneous	29	32	10	71
Disseminated	2	0	0	2
Mucocutaneous	1	2	4	7
Total	32	34	14	80

Based on the scale for the interpretation of F*_ST_* suggested by Wright (1978), the estimates showed significant genetic differentiation among the STRUCTURE clusters (Table 3). POP1 and POP3 showed moderate genetic differentiation (F*_ST_* = 0.1087), while POP2 showed great genetic differentiation with POP1 and POP3 (F*_ST_* = 0.1540 and 0.2028, respectively). POP1 had the highest average number of alleles per locus (23.3), while both clusters POP2 and POP3 were similar in mean number of alleles, being approximately five alleles per locus. Positive values of F*_IS_* were found for all clusters. F*_IS_* values for POP1 and POP3 were particularly high (Table 4). All loci were polymorphic for POP1 and POP2 and five (83.3%) of the six loci were polymorphic for POP3. The marker 6*pgd* was not polymorphic for POP3. In general, the new markers *hsp*70, *mdhnc* and *mdh*mt showed the highest number of alleles of 35, 40 and 44, respectively, in comparison to 15–30 alleles for the other three markers.

**Table 3:** Matrix of F*_ST_* values and corresponding p values for the three clusters identified in STRUCTURE of 86 Brazilian *L.* (*V.*) *braziliensis* strains.

p\F*_ST_*	POP1	POP2	POP3
POP1	0	0.1540	0.1087
POP2	0.0003	0	0.2028
POP3	0.0003	0.0003	0

F*_ST_* values can be found in the upper right triangle and p values with Bonferroni correction are reported in the lower left triangle of the matrix.

**Table 4:** Characterization of the three clusters found in the STRUCTURE analysis of *L.* (*V.*) *braziliensis* strains from Brazil.

Population	N	P	A	H_e_	H_o_	F*_IS_*
POP1	37	1.000	23.333	0.825	0.239	0.714
POP2	35	1.000	5.833	0.345	0.276	0.202
POP3	14	0.833	5.167	0.506	0.119	0.772

n – Sample size; P – Proportion of polymorphic loci; A – Mean number of alleles per locus; H_e_ - Expected heterozygosity; H_o_ – Observed heterozygosity; F*_IS_* – Inbreeding coefficient.

Results of the BURST and STRUCTURE analysis were found to be significantly associated (p<0.001) (Table 5). The majority (37/48) of unique sequences in the BURST analysis were forced into their own population (POP1) in the STRUCTURE analysis, representing mainly strains from the Amazon regions.

**Table 5:** Comparison of the results of the BURST and STRUCTURE analyses of the 86 *L.* (*V.*) *braziliensis* strains from Brazil.

Clonal Complex	Cluster			
	POP1	POP2	POP3	Total
CC1	0	31	3	34
CC2	0	0	2	2
CC3	0	0	2	2
Unique sequence	37	4	7	48
Total	37	35	14	86

Recombination events were detected by seven algorithms in RDP software (p<0.05). However, neither the beginning nor ending breakpoints could be identified, which may have resulted in recombinant misidentification. Nonetheless, one sample from Santa Catarina (185) and one sample from Bahia (IOC/L 2871) were indicated as potentially parental or recombinant. Thirty-one samples from Santa Catarina had sequences with partial evidence of the same recombination event.

**Figure 4:**
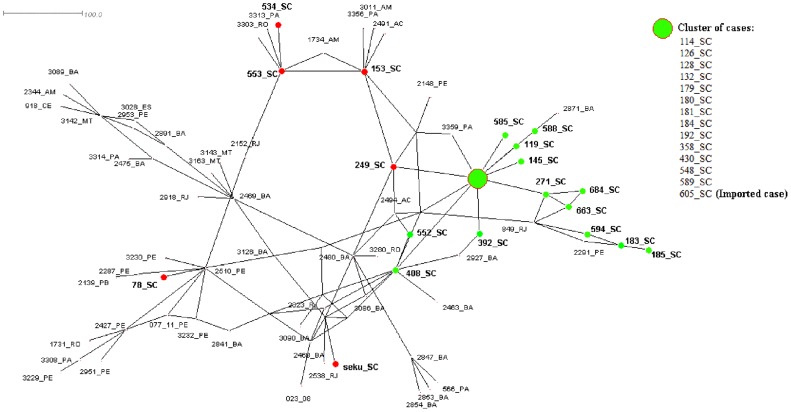
Median-joining network based on concatenated sequences of six gene fragments for 86 strains of *L.* (*V.*) *braziliensis* from Brazil, with case origin designated for strains from Santa Catarina. Green nodes represent autochthonous cases and red nodes represent imported cases.

The median-joining network was created from concatenated sequences of the six gene loci for the 86 strains of *L.* (*V.*) *braziliensis* from Brazil. Majority of Santa Catarina strains presented as an evident cluster. Other strains close to the Santa Catarina cluster were from Pernambuco (n = 2), Rio de Janeiro (n = 1), and Pará (n = 1). When the nodes of Santa Catarina strains were highlighted by case origin, all cases not clustered with the principal cluster were imported cases, with the exception of strain 605 (Figure 4). This 605 strain also was grouped within the main Santa Catarina CC and POP in both the BURST and STRUCTURE analyses.

**Figure 5:**
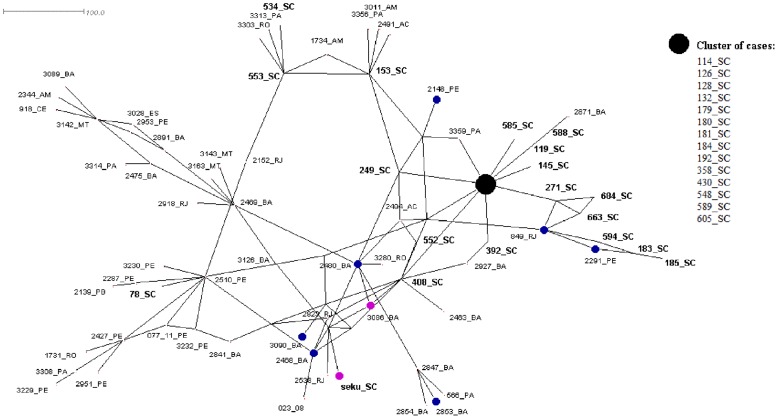
Median-joining network based on concatenated sequences of six gene fragments for 86 strains of *L.* (*V.*) *braziliensis* from Brazil, with clinical form of the case designated. Blue nodes represent mucocutaneous cases and purple nodes represent disseminated cases. Non-highlighted or black nodes signify cutaneous cases.

**Figure 6:**
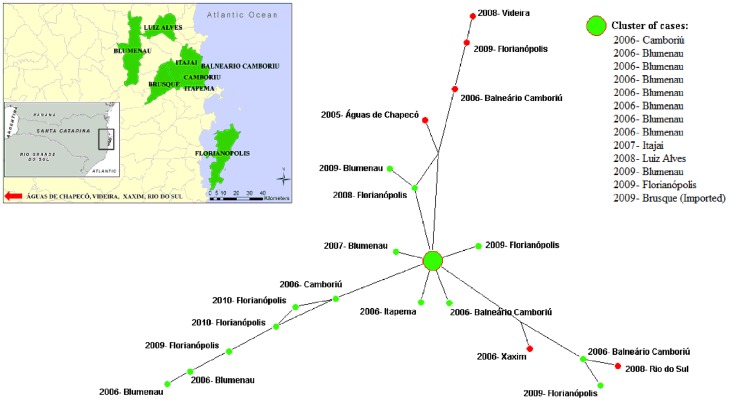
Reduced median-joining network based on concatenated sequences of six gene fragments for 33 strains of *L.* (*V.*) *braziliensis* from Santa Catarina, Brazil, with year, city of residence, and origin of case designated. Green nodes represent autochthonous cases and red nodes represent imported cases.

When the strains from cases of mucocutaneous and disseminated clinical form were highlighted, those from Bahia were clustered, while mucocutaneous cases from other Brazilian states appeared closer to the main cluster of Santa Catarina (Figure 5). When the median-joining network was reduced to only strains from Santa Catarina, the resulting network presented three principal branches. Marked by year and city of leishmaniasis case diagnosis, a main cluster can be observed in the center of the network, representing the epicenter of the outbreak which occurred in 2006 in the municipality of Blumenau (Figure 6). From this main epicenter, autochthonous cases branched separately, appearing to evolve over time and space to the neighboring municipality of the capital municipality of Florianópolis. The map in Figure 6 shows this main cluster of related Santa Catarina strains was distributed over a distance of 140 km in four years from Blumenau to Florianópolis.

## Discussion

Multilocus sequencing analysis (MLSA) was successful in detecting epidemiological patterns among *L.* (*V.*) *braziliensis* strains from twelve Brazilian states. Additionally, the technique was able to detect intra-species variation compatible with epidemiological characteristics within a specific outbreak focus, demonstrating the potential of this technique as a molecular tool for outbreak investigation.

In the BURST analysis, strains were found to group into three clonal complexes. Samples from the Amazon region presented largely as unique sequence types, demonstrating a proportionally higher level of heterogeneity in comparison to coastal states. This distinction is particularly apparent when compared to Santa Catarina. Since the BURST analysis permitted samples not to be grouped into a specific CC and remain as unique sequences, the STRUCTURE analysis was observed to force these unique sequences to form a genetic cluster. This was also evident in the significant association between the two analyses (p<0.001). POP1 was comprised of almost entirely unique sequence types. This high heterogeneity is characteristic of strains from the Amazon biome, as previously observed in other studies, and reflects the large variety of vectors and hosts in the region [10], [35], [36]. Furthermore, the emergence of *L.* (*V.*) *braziliensis* in the state of Santa Catarina, Brazil appears to be a recent event, given the high homogeneity observed among the analyzed strains. This conclusion is based on the assumptions of the Hardy-Weinberg equilibrium model of populations, which states if no evolutionary pressure mechanism, such as migration in or out of the population or mutation over a long period of time, is acting upon a given population, then the genetic frequencies will remain unaltered [37], [38]. Therefore, during the period from which the samples were collected during the outbreak, the strains were largely uninfluenced by outside strains, remaining as their own apparently unique population. This could also be caused by a specific transmission cycle in which other *Leishmania* strains or species were not easily incorporated. Specific vector-parasite relationships would remove the possibility of recombination given the major selective force on *Leishmania* populations occurs in the vector hosts during the development of the parasite [39].

MLSA utilizing the panel of six markers was able to distinguish epidemiological characteristics among *L.* (*V.*) *braziliensis* strains. In all three analyses (BURST, STRUCTURE, and median-joining), MLSA results were compatible with case origin evaluated in the epidemiological investigation of Santa Catarina strains. These results demonstrate the potential of this method for use in future outbreak investigations and surveillance. Despite being registered as an imported case in the epidemiological investigation, strain 605 was grouped within the main Santa Catarina cluster in all three analyses, pointedly suggesting this patient was most likely an autochthonous case. In such cases, the molecular characterization proved to be a more reliable and precise tool than the epidemiological interview to determine if a case acquired the infection locally or outside of a given region. Results also show the methodology possesses discriminatory power to differentiate imported and autochthonous cases at state macroregion levels. Knowledge on the origin of a case is important for predicting case outcome and treatment course, since several studies have shown a relationship between specific characteristics of the infecting parasite and geographical location with the outcome of the patient [9], [40], [41].

Along the same lines, MLSA showed a significant association between clusters in the STRUCTURE analysis and patient clinical form among the samples analyzed in the present study (p = 0.0296). However, the current study only included seven cases of the mucocutaneous form. A study involving a large representative sample of these cases with controls is necessary to validate these findings. Identification of a genetic marker of *Leishmania* virulence has not been identified at the present moment [42], and the identification of such a marker would have important clinical and pharmacological significance. Despite the limited number of samples in this study, this methodology could be promising for the identification of a specific *L.* (*V.*) *braziliensis* cluster predisposed to the mucocutaneous form, and therefore, warrants further investigation.

Recombination is often difficult to detect within species because of low inter-strain diversity and/or apparent low diversity due to inappropriate sampling [43]. However, RDP results of the present study were able to reveal recombination occurring between the *L. braziliensis* strains. This suggests the strains from Santa Catarina may be the result of a clonal expansion from a recombinant event, and the resulting strains then encountered proper conditions to propagate in the state. Previous studies on recombination, including a study on population genetics for inbreeding [44] and a previous MLSA phylogenetic study [18] specifically for *L. braziliensis*, were able to detect recombination signals as well. In these situations, homologous recombination may have been the responsible mechanism. This phenomenon also may have produced the well-structured clonal complexes in *Leishmania* in the present study which allowed for the epidemiological inferences to be made.

As no definitive set of markers for MLSA has been defined for the study of populations within a given species of *Leishmania*, the markers evaluated here could be defined as potential candidates in the panel used for this type of study. Interestingly, the three new markers, *hsp*70, *mdh*nc and *mdh*mt, were the most polymorphic of the six markers, suggesting their addition provided the increase in discriminatory power that allowed for intra-species differentiation. Taken together, these six markers provided adequate discriminatory power to answer epidemiological questions surrounding genetic clusters of a single species. An important benefit of MLSA is the ability to create and store sequences in an international database for global comparison of *Leishmania* species and strains [15]. The next step will be to determine the viability and discriminatory power of this six loci panel for other species of *Leishmania* and increase the number of markers and strains sequenced. Four of the markers (6*pgd*, *mpi*, *icd* and *hsp*70) have already proven to be discriminatory among species of the *Leishmania* subgenus *Viannia*, including *L.* (*V.*) *shawi*, *L.* (*V.*) *lainsoni*, *L.* (*V.*) *naiffi* and *L.* (*V.*) *guyanensis* [18], [21].

With the recent increase in development of genetic markers and new statistical methods for analyzing them, the choice of which software is most adequate to your specific analysis is becoming increasingly difficult. No definitive guidelines currently exist [45]. For this reason, we opted to evaluate our MLSA results from three different perspectives, using diverse software (BURST, STRUCTURE and Splitstree) to arrive at our inferences regarding the genetic structure among the *L.* (*V.*) *braziliensis* included in the present study. The BURST analysis, which is commonly used for MLSA of haploid organisms, such as bacteria, permitted a better comprehension of the genetic variability among the samples using conservative parameters for differentiating clonal complexes. As almost all Santa Catarina strains fit into one clonal complex and the remaining strains were mainly unique sequences, we can conclude the cluster in this state is highly homogeneous in comparison to other states. However, with over half of the strains not grouped in a clonal complex, comparison of genotypes with epidemiological factors was not possible. The STRUCTURE analysis forced all strains into a cluster, resulting in the grouping of all of these unique sequences into their own cluster. This phenomenon shows that, despite the high diversity among the samples from the Amazon region, strains from Santa Catarina continue to be genetically distinct from other Brazilian strains analyzed here. In other words, the diverse genetic clusters within POP1 of the Amazon region, as a whole, are still genetically more distinct from Santa Catarina strains than within themselves, as also shown by F*_ST_* and F*_IS_*. Interestingly, our study found high positive F*_IS_* values (high inbreeding coefficients) among the populations of *L.* (*V.*) *braziliensis*, which negates the hypothesis of strictly clonal reproduction among *Leishmania* species. High F*_IS_* values have also been observed in various MLMT studies for *Leishmania*, including a study on *L.* (*V.*) *braziliensis* in Bolivia and Peru [46], a study on *L.* (*L.*) *infantum* in Old World and New World strains [47] and a study on *L.* (*L.*) *donovani* in Ethiopia [48]. In these studies, possible explanations of these high F*_IS_* values were the presence of considerable inbreeding and/or sub-structuring of the population, reflecting a possible Wahlund effect.

Despite being too complex for comparing all strains among themselves, the median-joining network was the best visual representation for comparing Santa Catarina strains with all other strains from Brazil. This type of analysis is most applicable in an outbreak situation in which strains from a specific area can be compared to other reference strains, allowing for the distinguishing of imported cases and other epidemiological differences. Overall, until software capable of addressing specific genetic *Leishmania* characteristics, such as infrequent recombination, is created, use of all three types of genetic analyses can be used as an alternative to provide a robust MLSA analysis.

This information on the genetic variability of circulating strains is important for public health and control efforts. Considering drug resistance and complications in treatment have not been observed in Santa Catarina cases, control of leishmaniasis in Santa Catarina where the parasite strains are genetically homogeneous would be expected to be much more efficient than in regions where the parasite presents genetic heterogeneity and a more complex transmission cycle. This factor emphasizes the need for more urgent and active control methods to prevent further introduction of *Leishmania* strains and/or species, as well as geographical spread of the disease.

### Conclusion

MLSA revealed epidemiological patterns among *L.* (*V.*) *braziliensis* strains from twelve Brazilian states, even within the state of Santa Catarina where the strains presented extensive homogeneity. The addition of three markers, *hsp*70, *mdh*nc and *mdh*mt to the previously described panel of markers increased the discriminatory power of the technique, permitting the identification of three genetic clusters within *L.* (*V.*) *braziliensis* strains. All three analyses (BURST, STRUCTURE and median-joining network) provided a complementary and integral part in the interpretation of the MLSA results. When used in tandem with MLMT, these two methods could provide a more robust approach to the molecular epidemiology of leishmaniasis and increased validity of the population structure model. A prospective study design that seeks to include a representative sample of the patient population and active collection of their *Leishmania* strains is needed to validate this method as a molecular epidemiology tool. However, the present study has provided sufficient evidence of the effectiveness of this method for pursuing further validation of MLSA for leishmaniasis outbreak investigation.

## Supporting Information

Supporting Information S1 Summary of clonal complex and genetic cluster of strains by state.
(XLS)

Supporting Information S2 Strains by sequence type, with data on sex, clinical outcome, origin, and state of origin provided for Santa Catarina strains only. Doubled columns present alternative alleles for those sequences presenting ambiguous sites.
(XLS)

## References

[1] Reithinger R, Dujardin JC, Louzir H, Pirmez C, Alexander B, et al (2007) Cutaneous leishmaniasis. Lancet Infect Dis 7: 581–596. 17714672 10.1016/S1473-3099(07)70209-8

[2] Vos T, Flaxman AD, Naghavi M, Lozano R, Michaud C, et al (2012) Years lived with disability (YLDs) for 1160 sequelae of 289 diseases and injuries 1990–2010: a systematic analysis for the Global Burden of Disease Study 2010. Lancet 380: 2163–2196. 23245607 10.1016/S0140-6736(12)61729-2 PMC6350784

[3] Aguado M, Espinosa P, Romero-Mate A, Tardio JC, Cordoba S, et al (2013) Outbreak of cutaneous leishmaniasis in Fuenlabrada, Madrid. Actas Dermosifiliogr 104: 334–342. 23567452 10.1016/j.adengl.2013.03.005

[4] Desjeux P (2001) The increase in risk factors for leishmaniasis worldwide. Trans R Soc Trop Med Hyg 95: 239–243. 11490989 10.1016/s0035-9203(01)90223-8

[5] Varani S, Cagarelli R, Melchionda F, Attard L, Salvadori C, et al (2013) Ongoing outbreak of visceral leishmaniasis in Bologna Province, Italy, November 2012 to May 2013. Euro Surveill 18: 20530. 23929116

[6] Villinski JT, Klena JD, Abbassy M, Hoel DF, Puplampu N, et al (2008) Evidence for a new species of Leishmania associated with a focal disease outbreak in Ghana. Diagn Microbiol Infect Dis 60: 323–327. 18031968 10.1016/j.diagmicrobio.2007.09.013

[7] Salomon OD, Sosa-Estani S, Ramos K, Orellano PW, Sanguesa G, et al (2006) Tegumentary leishmaniasis outbreak in Bella Vista City, Corrientes, Argentina during 2003. Mem Inst Oswaldo Cruz 101: 767–774. 17160285 10.1590/s0074-02762006000700010

[8] Faucher B, Gaudart J, Faraut F, Pomares C, Mary C, et al (2012) Heterogeneity of environments associated with transmission of visceral leishmaniasis in South-Eastern France and implication for control strategies. PLoS Negl Trop Dis 6: e1765. 22880142 10.1371/journal.pntd.0001765 PMC3413717

[9] Marlow MA, da Silva Mattos M, Makowiecky ME, Eger I, Rossetto AL, et al (2013) Divergent profile of emerging cutaneous leishmaniasis in subtropical Brazil: new endemic areas in the southern frontier. PLoS One 8: e56177. 23457521 10.1371/journal.pone.0056177 PMC3572950

[10] Grimaldi G Jr, Tesh RB, McMahon-Pratt D (1989) A review of the geographic distribution and epidemiology of leishmaniasis in the New World. Am J Trop Med Hyg 41: 687–725. 2701633 10.4269/ajtmh.1989.41.687

[11] Banuls AL, Hide M, Prugnolle F (2007) Leishmania and the leishmaniases: a parasite genetic update and advances in taxonomy, epidemiology and pathogenicity in humans. Adv Parasitol 64: 1–109. 17499100 10.1016/S0065-308X(06)64001-3

[12] Schonian G, Kuhls K, Mauricio IL (2011) Molecular approaches for a better understanding of the epidemiology and population genetics of Leishmania. Parasitology 138: 405–425. 21078222 10.1017/S0031182010001538

[13] Gelanew T, Cruz I, Kuhls K, Alvar J, Canavate C, et al (2011) Multilocus microsatellite typing revealed high genetic variability of Leishmania donovani strains isolated during and after a Kala-azar epidemic in Libo Kemkem district, northwest Ethiopia. Microbes Infect 13: 595–601. 21382503 10.1016/j.micinf.2011.02.003

[14] Motoie G, Ferreira GE, Cupolillo E, Canavez F, Pereira-Chioccola VL (2013) Spatial distribution and population genetics of Leishmania infantum genotypes in Sao Paulo State, Brazil, employing multilocus microsatellite typing directly in dog infected tissues. Infect Genet Evol 18: 48–59. 23665466 10.1016/j.meegid.2013.04.031

[15] Schonian G, Mauricio I, Gramiccia M, Canavate C, Boelaert M, et al (2008) Leishmaniases in the Mediterranean in the era of molecular epidemiology. Trends Parasitol 24: 135–142. 18262469 10.1016/j.pt.2007.12.006

[16] Chicharro C, Llanes-Acevedo I, Garcia E, Nieto J, Moreno J, et al (2013) Molecular typing of Leishmania infantum isolates from a leishmaniasis outbreak in Madrid, Spain, 2009 to 2012. Euro Surveill 18: 20545. 23929179 10.2807/1560-7917.es2013.18.30.20545

[17] Seridi N, Amro A, Kuhls K, Belkaid M, Zidane C, et al (2008) Genetic polymorphism of Algerian Leishmania infantum strains revealed by multilocus microsatellite analysis. Microbes Infect 10: 1309–1315. 18755285 10.1016/j.micinf.2008.07.031

[18] Boite MC, Mauricio IL, Miles MA, Cupolillo E (2012) New insights on taxonomy, phylogeny and population genetics of Leishmania (Viannia) parasites based on multilocus sequence analysis. PLoS Negl Trop Dis 6: e1888. 23133690 10.1371/journal.pntd.0001888 PMC3486886

[19] Mauricio IL, Yeo M, Baghaei M, Doto D, Pratlong F, et al (2006) Towards multilocus sequence typing of the Leishmania donovani complex: resolving genotypes and haplotypes for five polymorphic metabolic enzymes (ASAT, GPI, NH1, NH2, PGD). Int J Parasitol 36: 757–769. 16725143 10.1016/j.ijpara.2006.03.006

[20] Zemanova E, Jirku M, Mauricio IL, Horak A, Miles MA, et al (2007) The Leishmania donovani complex: genotypes of five metabolic enzymes (ICD, ME, MPI, G6PDH, and FH), new targets for multilocus sequence typing. Int J Parasitol 37: 149–160. 17027989 10.1016/j.ijpara.2006.08.008

[21] Tsukayama P, Lucas C, Bacon DJ (2009) Typing of four genetic loci discriminates among closely related species of New World Leishmania. Int J Parasitol 39: 355–362. 18817779 10.1016/j.ijpara.2008.08.004

[22] da Silva LA, de Sousa Cdos S, da Graca GC, Porrozzi R, Cupolillo E (2010) Sequence analysis and PCR-RFLP profiling of the hsp70 gene as a valuable tool for identifying Leishmania species associated with human leishmaniasis in Brazil. Infect Genet Evol 10: 77–83. 19913112 10.1016/j.meegid.2009.11.001

[23] Garcia L, Kindt A, Bermudez H, Llanos-Cuentas A, De Doncker S, et al (2004) Culture-independent species typing of neotropical Leishmania for clinical validation of a PCR-based assay targeting heat shock protein 70 genes. J Clin Microbiol 42: 2294–2297. 15131217 10.1128/JCM.42.5.2294-2297.2004 PMC404633

[24] Tamura K, Dudley J, Nei M, Kumar S (2007) MEGA4: Molecular Evolutionary Genetics Analysis (MEGA) software version 4.0. Mol Biol Evol 24: 1596–1599. 17488738 10.1093/molbev/msm092

[25] Librado P, Rozas J (2009) DnaSP v5: a software for comprehensive analysis of DNA polymorphism data. Bioinformatics 25: 1451–1452. 19346325 10.1093/bioinformatics/btp187

[26] Feil EJ, Li BC, Aanensen DM, Hanage WP, Spratt BG (2004) eBURST: inferring patterns of evolutionary descent among clusters of related bacterial genotypes from multilocus sequence typing data. J Bacteriol 186: 1518–1530. 14973027 10.1128/JB.186.5.1518-1530.2004 PMC344416

[27] Spratt BG, Hanage WP, Li B, Aanensen DM, Feil EJ (2004) Displaying the relatedness among isolates of bacterial species – the eBURST approach. FEMS Microbiol Lett 241: 129–134. 15598523 10.1016/j.femsle.2004.11.015

[28] Pritchard JK, Stephens M, Donnelly P (2000) Inference of population structure using multilocus genotype data. Genetics 155: 945–959. 10835412 10.1093/genetics/155.2.945 PMC1461096

[29] Earl D, vonHoldt B (2012) STRUCTURE HARVESTER: a website and program for visualizing STRUCTURE output and implementing the Evanno method. Conservation Genetics Resources 4: 359–361.

[30] Jakobsson M, Rosenberg NA (2007) CLUMPP: a cluster matching and permutation program for dealing with label switching and multimodality in analysis of population structure. Bioinformatics 23: 1801–1806. 17485429 10.1093/bioinformatics/btm233

[31] Dieringer D, Schlötterer C (2003) microsatellite analyser (MSA): a platform independent analysis tool for large microsatellite data sets. Molecular Ecology Notes 3: 167–169.

[32] Lewis POZD (2002) Genetic Data Analysis: Computer program for the analysis of allelic data, Version 1.1.

[33] Martin DP, Lemey P, Lott M, Moulton V, Posada D, et al (2010) RDP3: a flexible and fast computer program for analyzing recombination. Bioinformatics 26: 2462–2463. 20798170 10.1093/bioinformatics/btq467 PMC2944210

[34] Huson DH, Bryant D (2006) Application of phylogenetic networks in evolutionary studies. Mol Biol Evol 23: 254–267. 16221896 10.1093/molbev/msj030

[35] Cupolillo E, Brahim LR, Toaldo CB, de Oliveira-Neto MP, de Brito ME, et al (2003) Genetic polymorphism and molecular epidemiology of Leishmania (Viannia) braziliensis from different hosts and geographic areas in Brazil. J Clin Microbiol 41: 3126–3132. 12843052 10.1128/JCM.41.7.3126-3132.2003 PMC165365

[36] Gomes RF, Macedo AM, Pena SD, Melo MN (1995) Leishmania (Viannia) braziliensis: genetic relationships between strains isolated from different areas of Brazil as revealed by DNA fingerprinting and RAPD. Exp Parasitol 80: 681–687. 7758549 10.1006/expr.1995.1084

[37] Jameson DL (1977) Benchmark Papers in Genetics 8. Evolutionary Genetics. Stroudsburg, PA: Dowden, Hutchinson and Ross.

[38] Weinberg W (1908) Über den Nachweis der Vererbung beim Menschen. Jahresh. Ver Vaterl Naturkd Württemb 64: 369–382.

[39] Lainson RS, J.J. (1987) Evolution, classification and geographical distribution. The Leishmaniases in Biology and Medicine; Peters WK-K, R., editor. London: Academic Press.

[40] Arevalo J, Ramirez L, Adaui V, Zimic M, Tulliano G, et al (2007) Influence of Leishmania (Viannia) species on the response to antimonial treatment in patients with American tegumentary leishmaniasis. J Infect Dis 195: 1846–1851. 17492601 10.1086/518041

[41] Souza AS, Giudice A, Pereira JM, Guimaraes LH, de Jesus AR, et al (2010) Resistance of Leishmania (Viannia) braziliensis to nitric oxide: correlation with antimony therapy and TNF-alpha production. BMC Infect Dis 10: 209. 20633260 10.1186/1471-2334-10-209 PMC2915995

[42] Hartley MA, Ronet C, Zangger H, Beverley SM, Fasel N (2012) Leishmania RNA virus: when the host pays the toll. Front Cell Infect Microbiol 2: 99. 22919688 10.3389/fcimb.2012.00099 PMC3417650

[43] Prugnolle F, De Meeus T (2010) Apparent high recombination rates in clonal parasitic organisms due to inappropriate sampling design. Heredity (Edinb) 104: 135–140. 19812614 10.1038/hdy.2009.128

[44] Mannaert A, Downing T, Imamura H, Dujardin JC (2012) Adaptive mechanisms in pathogens: universal aneuploidy in *Leishmania* . Trends Parasitol 28: 370–376. 22789456 10.1016/j.pt.2012.06.003

[45] Halkett F, Simon JC, Balloux F (2005) Tackling the population genetics of clonal and partially clonal organisms. Trends Ecol Evol 20: 194–201. 16701368 10.1016/j.tree.2005.01.001

[46] Rougeron V, De Meeus T, Hide M, Waleckx E, Bermudez H, et al (2009) Extreme inbreeding in Leishmania braziliensis. Proc Natl Acad Sci U S A 106: 10224–10229. 19497885 10.1073/pnas.0904420106 PMC2700931

[47] Kuhls K, Alam MZ, Cupolillo E, Ferreira GE, Mauricio IL, et al (2011) Comparative microsatellite typing of new world leishmania infantum reveals low heterogeneity among populations and its recent old world origin. PLoS Negl Trop Dis 5: e1155. 21666787 10.1371/journal.pntd.0001155 PMC3110170

[48] Gelanew T, Kuhls K, Hurissa Z, Weldegebreal T, Hailu W, et al (2010) Inference of population structure of Leishmania donovani strains isolated from different Ethiopian visceral leishmaniasis endemic areas. PLoS Negl Trop Dis 4: e889. 21103373 10.1371/journal.pntd.0000889 PMC2982834

