# Correction: Multilocus Sequence Analysis for *Leishmania braziliensis* Outbreak Investigation

**DOI:** 10.1371/journal.pntd.0002962

**Published:** 2014-05-28

**Authors:** The PLOS Neglected Tropical Diseases Staff

There is an error in Table 1. The primer sequence for Heat-shock protein 70 (hsp 70) should be:

**Table 1:** Amplicon size, analyzed sequence fragment length and primer sequence of the target regions for the six loci studied.

Locus	Amplicon size (bp)	Analyzed sequence length (bp)	Primer sequence (5’-3’)
6-*phosphogluconate dehydrogenase* (6*pgd*)	836	666	CTCAAGGAACATGAGCACGA TTGTCCTTGACTTGCTCACG
*Manose*-6-*phosphate isomerase* (*mpi*)	681	569	GGCAAGATGTATGCGGAGTT CTCCCCAGGAACCATCTGTA
*Isocitrate dehydrogenase* (*icd*)	1022	755	GAATCGGGAAGGAGATCACA CATCATAGCCCCAGAGAGGA
*Heat-shock protein* 70 (*hsp*70)	1022	896	GACGGTGCCTGCCTACTTCAA CCGCCCATGCTCTGGTACATC
*Malate dehydrogenase* mitochondrial (*mdh*mt)	821	666	TGCCGACCTCTTCCATATTC GAGTGAGGTGCGTCTTCACA
*Malate dehydrogenase* nuclear (*mdh*nc)	1010	803	TCACAACCGCAACTACGA CTACTCACGATAACGGCAGA

Forward: GAC GGT GCC TGC CTA CTT CAA, Reverse: CCG CCC ATG CTC TGG TAC ATC.
